# A qualitative study of clinic and community member perspectives on intervention toolkits: “Unless the toolkit is used it won’t help solve the problem”

**DOI:** 10.1186/s12913-017-2413-y

**Published:** 2017-07-18

**Authors:** Melinda M. Davis, Sonya Howk, Margaret Spurlock, Paul B McGinnis, Deborah J. Cohen, Lyle J. Fagnan

**Affiliations:** 1 Oregon Rural Practice-based Research Network (ORPRN), School of Medicine (Department of Family Medicine), School of Public Health, Oregon Health & Science University (OHSU), 3181 SW Sam Jackson Park Road Mailcode: FM, Portland, OR 97239 USA; 2 Oregon Rural Practice-based Research Network, Oregon Health & Science University, 3181 SW Sam Jackson Park Road, Mailcode: L222, Portland, OR 97239 USA; 3 Multnomah County School-Based Health Center Program, Oregon, Portland USA; 4 Greater Oregon Behavioral Health Inc, 309 East 2nd Street, The Dalles, OR 97058 USA; 5 Department of Family Medicine, Oregon Health & Science University (OHSU), 3181 SW Sam Jackson Park Road Mailcode: FM, Portland, OR 97239 USA; 6 Oregon Rural Practice-based Research Network, Department of Family Medicine, Oregon Health & Science University, 3181 SW Sam Jackson Park Road, Mailcode: L222, Portland, OR 97239 USA

**Keywords:** Practice change, Knowledge translation, Toolkits, Primary care, Community-based research, Implementation research, Practice facilitation, Qualitative methods

## Abstract

**Background:**

Intervention toolkits are common products of grant-funded research in public health and primary care settings. Toolkits are designed to address the knowledge translation gap by speeding implementation and dissemination of research into practice. However, few studies describe characteristics of effective intervention toolkits and their implementation. Therefore, we conducted this study to explore what clinic and community-based users want in intervention toolkits and to identify the factors that support application in practice.

**Methods:**

In this qualitative descriptive study we conducted focus groups and interviews with a purposive sample of community health coalition members, public health experts, and primary care professionals between November 2010 and January 2012. The transdisciplinary research team used thematic analysis to identify themes and a cross-case comparative analysis to explore variation by participant role and toolkit experience.

**Results:**

Ninety six participants representing primary care (*n* = 54, 56%) and community settings (*n* = 42, 44%) participated in 18 sessions (13 focus groups, five key informant interviews). Participants ranged from those naïve through expert in toolkit development; many reported limited application of toolkits in actual practice. Participants wanted toolkits targeted at the right audience and demonstrated to be effective. Well organized toolkits, often with a quick start guide, with tools that were easy to tailor and apply were desired. Irrespective of perceived quality, participants experienced with practice change emphasized that leadership, staff buy-in, and facilitative support was essential for intervention toolkits to be translated into changes in clinic or public -health practice.

**Conclusions:**

Given the emphasis on toolkits in supporting implementation and dissemination of research and clinical guidelines, studies are warranted to determine when and how toolkits are used. Funders, policy makers, researchers, and leaders in primary care and public health are encouraged to allocate resources to foster both toolkit development *and* implementation. Support, through practice facilitation and organizational leadership, are critical for translating knowledge from intervention toolkits into practice.

## Background

Policy makers, funders, and researchers express concern that scientific discoveries are not being translated into primary care or public health settings [1–3]. Studies indicate it takes 17 years for 14% of research evidence to reach practice, and the implementation of evidence-based interventions is often incomplete or ineffective [4, 5]. Members of medical and public health communities are making substantial efforts to address this knowledge translation gap.

Intervention toolkits are increasingly requested by funding agencies as research products that can be used to support the translation of evidence-based practices into diverse real-world settings. A search for the keyword “toolkits” on Ovid Medline yields 1753 hits between 1946 to 2014, with 53.6% (*n* = 939) occurring after 2010 and only 3.9% (*n* = 69) before 1999. In our practice-based research network, the Oregon Rural Practice-based Research Network (ORPRN), we received funding from three distinct entities from July 2009 through 2011 to produce intervention toolkits from project findings.^1^ Despite the burgeoning emphasis on toolkits as a mechanism for knowledge translation, there is limited empirical research identifying characteristics of effective intervention toolkits and their implementation [6–9].

While there is not a uniformly accepted definition for intervention toolkits, the Agency for Healthcare Research and Quality (AHRQ) defines a toolkit as “an action-oriented compilation of related information, resources, or tools that together can guide users to develop a plan or organize efforts to conform to evidence-based recommendations or meet evidence-based specific practice standards” [10]. Toolkits are designed to lead users through the process of developing a plan and organizing efforts to accomplish specific tasks by providing action-oriented recommendations and tools (e.g., surveys, guidelines, or checklists) [6, 10]. For example, the American Academy of Family Physicians created the “Americans in Motion-Healthy Interventions (AIM-HI) to Change Toolkit,” which consists of a practice manual, fitness posters, screening inventories, fitness prescription pads and other resources, to help primary care clinicians create a culture of fitness within their practice [11]. A Google search on “health intervention toolkits” yields resources to help potential users improve health literacy [12], redesign care delivery in hospital systems [13] and primary care settings [14], or to build healthy communities [15].

Despite the proliferation of intervention toolkits, a paucity of research explores when toolkits are utilized, how they are used, or what characteristics make them effective [6–10, 16]. Existing published and grey literature shows substantial variability in the content, underlying evidence base, and format across health intervention toolkits [7]. Many toolkits have not been rigorously evaluated; a recent systematic review concluded that toolkits should provide evidence of their potential to impact health and practice-change outcomes [8]. Very few studies, however, include an assessment of toolkit fidelity or implementation outcomes, nor have studies examined the effectiveness of different components or characteristics of toolkits or the contextual factors that contribute to successful use in practice [7, 8]. Thus the current research on intervention toolkits does not provide adequate evidence to guide product development or to inform successful application in practice.

As a first step in addressing this gap in the research literature and helping research teams build effective intervention toolkits, we used qualitative methods to determine what stakeholders from primary care and community settings (a) desired in intervention toolkits and (b) identified as contextual factors necessary to support the application of intervention toolkits in practice.

## Methods

We used a qualitative descriptive approach as this method is suited to obtaining answers to questions of key importance to practitioners and policy makers by gaining knowledge of participants’ experiences with a specific topic [17–19]. Qualitative description provides a robust summary of events, specifically by staying close to the words and “everyday language” used by participants [18]. Data collection and analysis was conducted by a transdisciplinary team with expertise in implementation science, qualitative methods, rural health, community health development, and participatory research in primary care and public health settings. The Oregon Health & Science University Institutional Review Board approved this study (IRB # 6837).

### Setting and participants

Data were collected as part of larger research initiative in rural Oregon to link rural primary care clinics with community-based resources for obesity management [20]. Specifically, we invited individuals associated with participating primary care practices, community-based health coalitions, our expert advisory panel of public health leaders, and primary care clinician members of the ORPRN steering committee to participate. We distributed information about the study and an invitation to participate by mail or email; interested participants could participate in a focus group or in a one-on-one interview. Our objective was to engage a purposive sample of participants by role (primary care, community/public health), gender (male, female), and who displayed a breadth of experience utilizing intervention toolkits and/or supporting change in practice and community settings.

### Data collection

**Fig. 1:**
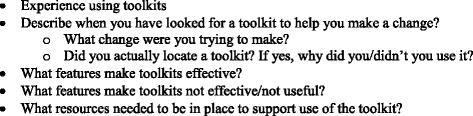
Relevant domains of semi-structure interview guide

One author (PM), ORPRN’s Director of Community Health, Quality, and Practice Development, facilitated all sessions using a semi-structured interview guide, see Fig. 1. The interview guide was developed by our transdisciplinary team, tailored to participant category (i.e., primary care; community/public health), and refined concurrently with data collection when areas warranting additional exploration emerged. The facilitator (PM) had more than 25 years of experience working with rural primary care and community-based stakeholders; he was experienced in qualitative methods and committed to helping rural partners improve regional health and health care delivery through community health development, quality improvement, and research.

We conducted focus groups and individual interviews to accommodate participant availability. Sessions were held in a private meeting space at the participant’s place of work or in the community and lasted approximately 1 h in duration. Primary care and community-based interviews occurred separately, except in regions where participants requested meeting jointly to facilitate the larger initiative (i.e., linking clinics with community-based resources). All participants provided written informed consent and completed a brief demographic survey prior to each session. Sessions were audio recorded and transcribed verbatim; a trained research assistant took detailed field notes during all sessions to describe non-verbal communication and to capture key content when audio recording was not possible. From November 2010 to January 2012 we conducted 18 qualitative sessions, including 13 focus groups and five key-informant interviews with 96 total participants. We did not document rates of non-participation by invited members because we did not want this to negatively impact an individual’s standing in their organization. We collected data until saturation was reached [21, 22].

### Data analysis

The authors entered data from the demographic surveys into REDCap (Research Electronic Data Capture) [23] and imported qualitative data into Atlas.ti (Version 7.0, Berlin) for management and analysis. Concurrent with data collection, two authors (MMD, SH) used thematic analysis to classify emergent themes: reading transcripts independently, coding key phrases, and meeting to discuss codes and identify emergent themes [24]. A cross-case comparative analysis by participant role was used to examine similarities and differences among perspectives. We refined emerging themes during two retreats engaging additional transdisciplinary team members (PM, DC, LJF). Preliminary findings were shared with workshop participants at the 2011 AHRQ Annual Practice-based Research Network (PBRN) Conference as a form of member checking. We used this information to help finalize our emergent themes.

## Results

We conducted eighteen sessions with 96 participants from primary care (*n* = 54, 56%) and community settings (*n* = 42, 44%; includes community health coalition members and public health experts), see Table 1. Mean participant age for clinic and community participants combined was 48.8 years (Range: 19 – 79 years) and 81% were female. Community participants represented diverse geographic regions of rural Oregon and national public health experts. Clinic participants drew from seven public and private rural primary care practices (including Federally Certified Rural Health Clinics and Federally Qualified Health Centers) and clinician members of the ORPRN steering committee.

**Table 1:** Demographic and professional roles of clinic and community participants

Clinic participants (*N* = 54)	Percentage (*n*)
Demographics	
Gender (female)	74%
Mean age in years (range)	45.0 (19 – 67)
Roles/training	
Administration	19% (10)
Back office staff (nursing, medical assistants)^a^	35% (19)
Clinician (MD, DO, PA, NP)	35% (19)
Front office staff (reception)	11% (6)
Community Participants (*N* = 42)	
Demographics	
Gender (Female)	91%
Mean age in years (Range)	53.6 (23 – 79)
Roles/training	
Public health/health dept.	19% (8)
Medical clinic	5% (2)
Weight loss/physical activity agency	19% (8)
Hospital staff	12% (5)
Schools/school district	10% (4)
Other committee, commission, or non-profit	21% (9)
Other^b^	14% (6)

^a^Two participants reported playing dual medical assistant/administration roles

^b^Other community roles included: Bank (*n* = 1); Parks & Recreation (*n* = 1), not specified (*n* = 4)

Four descriptive themes emerged. The first three related to participant understanding of toolkits, including: definitions, use (or lack thereof), and characteristics perceived to be effective. A fourth theme emerged related to participants concerns that having an intervention toolkit alone did not ensure a practice or community-based program would change.

### Toolkit definitions

Despite prompts about “toolkits,” participants discussed resources that they were familiar with. Community members lacking direct experience with intervention toolkits highlighted their work with educational materials, training curricula, or instruction manuals. Clinic participants described materials from pharmaceutical sales representatives.

In defining intervention toolkits, participants indicated that they consisted primarily of practical, action-oriented materials and templates and are distinct from other resources that simply provide descriptive details on a topic area or concept. This distinction was illustrated by the following exchange during a focus group:

*Clinic Office Manager (P39): …guidebook(s) provide suggestions of how you might proceed and a toolkit tends to be more hands on, providing actual forms to help you get there….*

*Community Representative (P36): Yeah...a toolkit [has] materials and tools where I can actually follow certain steps and use these tools and put something into action… I like toolkits because they’re more practical and action oriented.*

Participants suggested that what potential users want in an intervention toolkit may depend on how much they know about a topic, or what their goals are. Those new to an area may want general information, while those who are ready to make changes may prefer specific resources (or tools) for immediate application. A public health expert (P83) stated, “*The pieces you use [in a toolkit] depend on where you are in the overall process. If you have started the process and are looking for answers to help the process along you only go to those areas of the toolkit.”*

### Toolkit usage

Although some participants reported using intervention toolkits to make personal, organizational, or practice-level changes, many reported limited application in their practice or community settings. A community member (P5) commented, *“[toolkits] get pretty dusty sitting on the shelf”* and a clinician (P53) who hadn’t used a toolkit stated, *“[Even if I had a toolkit] I probably wouldn’t read it.”* Some participants indicated that intervention toolkits and other resources often end up discarded despite good intentions. As another clinician (P31) stated, “*I just went through my mailbox and the garbage can was full.... I made a pile [of resources (e.g., toolkits, educational materials)] to take home for my spare time reading which will go in the recycle bin if I haven’t read it within a month and the rest of it will go into the garbage can.”*

Participants with experience using toolkits noted that first impressions frequently dictated whether or not the resource was used. A public health expert stated, “*....I’m going to evaluate a toolkit pretty quickly [to determine] whether I’m going to use it or not....I need something short that I’m going to want to go into deeper. That will get me to look at the full toolkit.”* A community member (P36) who reported substantial experience developing intervention toolkits noted that she reviews a toolkit’s summary to evaluate potential use: “*I like a summary at the beginning that tells me what this is about and what it offers. Then I can decide quickly if I continue or if this is not for me.”*

### Characteristics of effective toolkits

Emergent themes around the characteristics of effective intervention toolkits focused on the importance of specifying the target audience, presenting materials that were tested and effective, producing a brief resource with high functionality, and having access to the toolkit in multiple formats (online, printed) with easy to tailor tools.

#### Specify the target audience

Participants preferred toolkits targeted to the right audience, whether clinicians, practice staff, practice facilitators [25], or public health officials. The end users should be identified early in a toolkit, to foreshadow what content would be included. Some clinician participants responded positively to intervention toolkits targeted to the whole practice rather than to individual clinicians, indicating that individual clinicians already bear the brunt of both supporting practice change and delivering routine patient care. One clinician (P37) stated,

*It’s always so hard when I get things pointed at me… ‘Manage COPD, do spirometry, encourage smoking cessation, and get the patient exercising’…. I like that a toolkit would speak to [all members of the clinic]. That you could find a champion who was maybe a receptionist, a phone operator, or a medical assistant [to help implement the change].*

#### Tested and Effective

Participants preferred resources that had been tested and demonstrated to work. A clinic member (P31) commented, “*I want to know that somebody’s actually tested out the toolkit so that when you do it, it works...Because if it doesn’t, then I’m not going to go to any part of that toolkit. I’m going to toss it out.”*

#### Brevity with high functionality

Generally, participants indicated that they preferred a toolkit that was *“short”* or *“very short”* if possible. However, further analysis indicated that document organization and ease of use were most essential features of intervention toolkits. Participants reasoned that potential users were busy and had limited time to apply toolkits to make change. A community member (P78) stated, “*I want a toolkit that’s simplistic and not overly wordy…You’re dealing with people who don’t have a lot of time to really read through a large document. If I can glean the information out of it easily and it’s step by step, I’m more likely to use it*.” Because participants preferred intervention toolkits to help accomplish a goal or task, they indicated that elaborate details may be unnecessary as highlighted by the following exchange by clinic participants:

*Participant 16: [I want a toolkit that is] not too wordy...get to the point. It’s like I didn’t ask to build the clock, I just asked what time was it…*

*Participant 18: More like outline format, as opposed to verbiage....Where you can look at bullet points, you can find any topic heading and go straight there without reading everything in the toolkit. [other clinic members make sounds of agreement]*

A table of contents, index, and ‘quick start guide’ (e.g., materials included with new electronic devices like mobile phones), were identified as helpful features for toolkits. These elements would allow potential users to identify relevant sections and tools and to skip content that was not pertinent to the current goal at hand. One community member (P70) commented:

*[I want] a good index…have it broken out. I use toolkits probably two or three times a week and sometimes I just want a sample to go look at and take pieces of, to share, or other times I want something to read. So have a good index so people can take parts they want and leave the stuff they don’t need.*

#### Multiple Formats with Easy to Tailor Tools

Participants desired toolkits in multiple formats, including web-based and printed versions. Regardless of format, participants wanted tools and templates that could be immediately applied or easily tailored to suite the local setting. A clinic member (P39) stated, “*I like specific examples, particularly if I’m looking for policies or procedures…It’s good to have a tool that you can take out and use parts of....I hate recreating the wheel every single time we do something.”* A public health expert (P83) commented, “*I like tools I can customize to my community or practice – such as by adding our logo.”*

### An intervention toolkit does not equate with actual practice change

Regardless of perceived toolkit quality, participants noted that the will, interest, and resources of the potential user and the organization critically affected implementation in practice. One public health expert (P85) stated:

*You’re not going to [accomplish something] by putting some words on a piece of paper and throwing some tools at [a potential user], right? If [they] don’t have will, then [a toolkit] is not going to help out anyway... But if they have will but don’t know how to execute, that’s where a toolkit can help.*

A tension emerged between the desire for effective intervention toolkits and the perceived level of resources required to make a change to clinical or public health practices. This tension was especially strong for participants who displayed greater experience in supporting practice and organizational transformation initiatives (e.g., public health experts, ORPRN steering committee members). These participants noted that support for the change initiative may be more important than having a “good” intervention toolkit. This perspective was particularly salient for the clinician members of the ORPRN steering committee, many of whom have had extensive experience leading practice change. One ORPRN steering committee clinician (P93) commented, “*I think [the toolkit] would be easy to use. I think the bigger issue is finding the time and energy to implement [the change] and to get staff buy-in. No matter how good the toolkit is, unless it is used correctly it won't help solve the problem.”* Another ORPRN steering committee clinician member (P90) stated:

*I appreciate the nuts and bolts, how-to of the toolkit. The harder part is the practical. Who do you have do this and with what resources? Having instructions is different than having someone knowledgeable to help make the change. Toolkits can be helpful, but also intimidating…they’re different than working with a practice facilitator or other another clinic that’s done it. It’s different than having a cheerleader in the practice to actually help you make the change.*

## Discussion

This qualitative study involving experienced and prospective users of health intervention toolkits from both primary care and community/public health settings identified characteristics of effective toolkits and factors that impacted the application of toolkits into practice. Participants noted that toolkits were distinguished by providing practical, action-oriented instruction and resource templates that could be used to achieve specific goals and outcome objectives. Participants preferred that intervention toolkits that specified the target audience (e.g., staff, clinicians), were tested to demonstrated effectiveness, displayed high functionality (e.g., well organized, searchable), and had tools that were easy to apply and readily modifiable regardless of toolkit format (e.g., print, online). Many participants wanted toolkits that were brief and direct, noting that end users were unlikely to have time to navigate large documents. Importantly, participants experienced with leading change in primary care and community-based settings noted that having access to an intervention toolkit does not equate with implementation in practice. These individuals emphasized the need for the target users to be interested in the toolkit topic as well as having resources in the form of leadership/organizational support, staff buy-in, and having someone to help translate toolkit content into practice.

Our participants, like many grant funders, noted that toolkits may provide actionable information to translate evidence-based practices into clinic and community settings. Likewise, many of the desired characteristics identified by our study participants are recommended in the limited published research literature on toolkits [6, 10, 16]. However, our findings indicate that application of intervention toolkits in real-world settings may be dependent on contextual factors that supersede toolkit quality and design. This finding is echoed by the paucity of research on this topic. In fact, over 15 years ago Crabtree and colleagues conducted a comparative case study of eight primary care practices that *purchased* Put Prevention into Practice (PPIP) toolkits [26]. The authors found that the toolkits were not used as problems frequently occurred with implementation and a ‘one size fits all’ intervention was inadequate to address the different organizational needs and existing office structure of diverse primary care practices. They concluded that, “just as knowledge alone is insufficient to change physician behavior, the tools provided in a [tool]kit are unlikely to alter established practice patterns.” [26] Evidence from the Study To Enhance Prevention by Understanding Practice (STEP-UP) clinical trial also indicated that tailoring interventions to fit the evolving needs of the clinical practice environment may contribute to the long-term sustainability of improvement efforts [27, 28]. Monroe (2000) emphasized the importance of providing adaptable toolkit materials that can be used to tailor different solutions to similar problems based on the local context [16]. More recently, Nowalk and colleagues evaluated a toolkit to implement standing order programs (SOPs) for influenza and pneumococcal vaccinations in adults, concluding that “additional strategies” and “additional resources” may be needed to assist practices in adopting and sustaining SOPs [29].

A theme emerging in this study, and anecdotal evidence from the authors’ cumulative experience supporting quality improvement and implementation and dissemination research in primary care and community settings, highlights the critical need for personnel to support the translation of intervention toolkits into practice. Practice facilitation, which is the use of organization development, quality improvement, and engagement skills by a trained health professional to support system change and to help practices build capacity to implement improvement initiatives [25, 30], may be one implementation support strategy that can be used as an adjunct to toolkit provision. Recently, Fernald and colleagues (2015) found that while practices were able to use a toolkit to begin improving laboratory testing processes, practice facilitation or other support was needed for clinics to achieve their quality improvement aims [9]. A systematic review indicating primary care practices are 2.8 times more likely to adopt evidence-based guidelines with practice facilitation provides further evidence that support is needed for making practice change [31].

This need for toolkit support was echoed by participants at the AHRQ PBRN Workshop on “Building a Toolkit that Gets Used.” One audience member emphasized that an implementation specialist may be necessary to move from the Type 2 thought processes used to produce toolkits (e.g., those which are deliberate, explicit, effortful and intentional) to the Type 1 thinking that shapes most behaviors (e.g., those that are unconscious, automatic, contextual, speedy) [32]. The importance of exploring factors associated with research translation is highlighted by a recent funding announcement from the NIH stating: “Implementation research studies should distance from prior assumptions that empirically-supported interventions can be transferred into any service setting without attention to local context and that a unidirectional flow of information (e.g., publishing a guideline) is sufficient to achieve practice change [33].” Like implementation of clinical guidelines, applying intervention toolkits in practice may require active review, facilitation, and tailoring for use across diverse real-world settings. Despite a growing body of evidence, facilitated support is just beginning to be identified as a standard requirement for translating intervention toolkits into real-world practice and community settings [34]. Providing the support necessary to translate intervention toolkits into practice may require infrastructure resources beyond what is readily available to clinics or public health organizations operating on thin financial margins.

This study has a few notable limitations. First, most participants resided in rural Oregon. Clinics and community partners in rural regions may have differential access to resources or staff to support transformation initiatives. Second, there is not currently an accepted definition for “toolkit” in the health and health care fields. This was apparent in our findings as clinic and community participants had varied understanding of what constitutes an “intervention toolkit”; those with more limited exposure to toolkits often reflected on their knowledge of educational curricula or other training resources. Finally, this study was not designed to determine if participant comments varied based on the quality or content of toolkits they had worked with in the past. Engaging participants from different regions, with more experience using high-quality toolkits, or with greater exposure to quality improvement infrastructure may have led to the identification of different characteristics and needs. Additional research is needed to explore if opinions regarding toolkit length are mediated by how committed the user is in relation to the change, the perceived value of the change relative to the effort required, or the functionality/quality (or lack there-of) of the toolkit.

Despite these limitations, study findings portray preferences from a broad sample of potential intervention toolkit end users that can inform future research and quality improvement efforts in primary care and community-based settings. Our team utilized various approaches to ensure qualitative validity, including using an ethical approach to data collection, having multiple reviewers participate in the analysis, and engaging informal member checking [21, 35]. Unlike other studies, the current research involved participants with substantial diversity by role (e.g., primary care clinicians, staff members, public health leaders) and experience with toolkits and practice change. Preliminary themes resonated with approximately 25 national experts in implementation research and practice change who attended the 2011 AHRQ PBRN conference workshop on building toolkits that get used. Similar perceptions from diverse stakeholders and national experts in research translational suggest that results are generalizable to other regions and organizational settings. Future research should determine if these perceived characteristics are associated with toolkit effectiveness and utilization in practice. Viewing primary care practices and communities as complex adaptive systems, where components (agents) are linked by relationships that self-organize and interact in non-linear ways over time [36, 37], suggests the need for additional studies to clarify what resources are necessary to support toolkit implementation across diverse contextual settings (e.g., practices with high versus low quality improvement infrastructure or adaptive reserve). Tailoring toolkit support to the local setting may contribute to improving health outcomes and reducing the research translational gap.

## Conclusion

This study provides research evidence regarding the characteristics community, public health, and primary care professionals desire in intervention toolkits and what may facilitate their application in practice. Participants preferred toolkits targeted at the right audience, demonstrated to be effective, and containing tools that were easy to tailor and apply. Toolkits with a quick start guide or table of contents that directed users to key, relevant content were desired. Regardless of toolkit quality, infrastructure to support the application of intervention toolkits in practice was critical. Organizational buy-in and support from trained practice facilitators or quality improvement experts may be necessary to enable translation of evidence-based intervention toolkits into practice. Funders, policy makers, and community and practice leadership should consider allocating resources to support intervention toolkit implementation in addition to toolkit development.

## Abbreviations

AHRQ: Agency for Healthcare Research and Quality
NIH: National Institute of Health
ORPRN: Oregon Rural Practice-based Research Network
PBRN: Practice-based Research Network.

## Footnotes

1 Funding sources and toolkit titles: The Informed Medical Decisions Foundation – Integrating Decision Aids into Rural Primary Care to Support Shared Decision Making; the NIH National Center for Research Resources – Transitioning Community Health Improvement Partnerships (CHIP) into Community Health Improvement and Research Partnerships (CHIRP), and the Agency for Healthcare Research and Quality – Linking Primary Care Practices with Community-based Resources for Obesity Management.
